# DNA methylation-based health predictors: advances, applications, and perspectives

**DOI:** 10.1080/17501911.2025.2550932

**Published:** 2025-08-26

**Authors:** Zongli Xu

**Affiliations:** Biostatistics & Computational Biology Branch, National Institute of Environmental Health Sciences, NIH, Research Triangle Park, NC, USA

**Keywords:** DNA methylation, epigenetics, DNAm predictor, biological aging, epigenetic clock

## Abstract

DNA methylation (DNAm) has emerged as a powerful and dynamic biomarker for predicting health outcomes, biological aging, and disease risk. Unlike static genetic variants, DNAm is dynamic and influenced by environmental, lifestyle, and pathological factors, making it highly suitable for applications in personalized medicine. This review provides a comprehensive synthesis of recent advances in DNAm-based predictors, including epigenetic clocks, exposure biomarkers, disease risk models, and trait-specific estimators. We describe the diverse methodological frameworks underpinning these predictors, such as penalized regression, surrogate modeling and deep learning. We discuss their performance across various preprocessing strategies and study populations. Additionally, we highlight clinical and research applications, ethical considerations, and emerging challenges, such as issues of reproducibility, tissue specificity, population generalizability, and interpretability. Looking forward, we explore future directions emphasizing artificial intelligence, multiomics integration, and longitudinal modeling. By critically assessing current limitations and technological innovations, this review outlines a roadmap for advancing the development, validation, and responsible implementation of DNAm-based health predictors.

## 1. Introduction

DNA methylation (DNAm), the addition of a methyl group to the 5′ position of cytosine residues within CpG dinucleotides, is a crucial epigenetic modification that regulates gene expression, maintains genomic stability, and defines cellular identity. Unlike static genetic variants, DNAm patterns is highly dynamic and responsive to age, environmental exposures, and disease processes, making it an ideal candidate for biomarkers that reflect individual health states and trajectories [1,2].

Technological advances, particularly the development of Illumina’s 450K and EPIC BeadChips [3,4], and whole-genome bisulfite sequencing [5], have enabled high-throughput measurement of methylation across hundreds of thousands of CpG sites. These platforms have fueled large-scale epigenome-wide association studies (EWAS), revealing reproducible associations between DNAm and diverse exposures, traits, and disease risks [6,7].

One major outcome of this research has been the development of DNAm-based health predictors, which include epigenetic age clocks [8,9], disease risk estimators [10,11], and methylation-based exposure proxies [12–14]. These models range from simple linear regressions using a few CpGs to complex machine learning algorithms and composite scores incorporating intermediate phenotypes. Newer clocks like PhenoAge [10], GrimAge [11], DunedinPACE [15], and deep learning models like DeepMAge [16] and AltumAge [17] have further advanced the predictive power and functional relevance of DNAm-based biomarkers.

DNAm-based predictors are built on the principle that methylation levels at specific CpG dinucleotides correlate with biological outcomes, such as chronological age, disease risk, or environmental exposures [8,13]. Unlike single CpG sites, which often have limited predictive power due to measurement variability and small effect sizes, combinations of multiple CpGs provide robust and stable predictions by capturing complex epigenetic patterns. These CpGs are typically identified through EWAS, which detect sites with significant associations to the target outcome (e.g., aging, smoking status) across large cohorts. Statistical and machine learning methods, such as penalized regression (e.g., elastic net used in Horvath and GrimAge clocks) or deep learning (e.g., DeepMAge), are then applied to select a subset of CpGs and construct predictive models [8,16]. These models integrate methylation data from multiple CpGs to generate accurate health predictors, adaptable to various tissues and populations.

This review synthesizes recent developments in the design, application, and interpretation of DNAm-based health predictors. We highlight [1]: methodological advances spanning elastic net regression, surrogate modeling, principal component and deep learning [2]; diverse applications including biological age estimation, disease risk prediction, and lifestyle/exposure biomarkers [3]; technical and interpretive challenges such as batch effects, tissue specificity, generalizability, and causality; and [4] future directions emphasizing AI integration, multi-omics modeling, and clinical translation. As methylation profiling becomes increasingly standardized and accessible, these predictors are poised to play a transformative role in precision medicine, population health, and aging research.

## 2. DNAm-based health predictors

DNAm-based predictors are powerful tools for estimating age, disease risk, environmental exposures, and physiological traits. Table 1 summarizes key categories, representative predictors, and supporting references.

**Table 1.** Summary of DNA methylation-based predictors by category.

Category	Predictor	Note	Ref
Chronological Age Clocks	Horvath Clock	Pan-tissue clock	[8]
	Hannum Clock	Based on individuals aged 19–101	[9]
	cAge	Blood and saliva – based chronological age	[18]
	Epigenetic Age	Blood and saliva – based chronological age	[19]
	PedBE	Pediatric buccal cell, ages 0–20	[20]
	GACPC,GARPC,GARRPC	Gestational Age, placental tissue	[21]
	BohlinGAge	Gestational Age in days, cord blood	[22]
	KnightGAge	Gestational Age in weeks, cord blood	[23]
	DeepMAge	Neural network based on age predictor	[16]
	AltumAge	Pan-tissue clock based on neural network	[17]
	DNAm-metabolic clock	Use DNAm-derived metabolite proxies	[24]
Biological Age Clocks	PhenoAge	Predict biomarker inferred lifespan	[10]
	GrimAge	Use plasma protein and smoking proxies	[11]
	Zhang10CpG	Predict all-cause mortality	[25]
	Frailty Clock	Epigenetic frailty risk score	[26]
	bAge	Use DNAm proxies for plasma proteins	[18]
	DNAmFitAge	Use DNAm proxies for physical fitness	[27]
	DNAmTL	Estimate of telomere length	[28]
Pace-of-Aging Clocks	DunedinPACE	Pace of aging using longitudinal cohort	[15]
Disease Risk Predictors	Epi proColon	Colorectal cancer detection	[29]
	Galleri	Multicancer early detection assay	[30]
	MRS for CHD	Coronary heart disease risk	[31]
Lifestyle Exposure Biomarkers	EpiSmokEr	Smoking status	[12]
	McCartney Smoking Score	Cumulative smoking in pack-year	[13]
	Alcohol Predictor	Alcohol consumption predictor	[14]
Metabolic and Anthropometric	BMI,Body Fat, HDL Waist-to-Hip Ratio	Anthropometric traits prediction	[13]
Physical Function and Fitness	DNAmGrip, DNAmGait DNAmFEV1, DNAmVO2max DNAmFitAge	biological age indicator that incorporates physical fitness	[27]
Stem Cell Division/Mitotic History	pcgtAge	Based on polycomb group target CpGs	[32]
	MiAge	Based on mitotic informative CpGs	[33]
	TNSC, TNSC2	Imply both aging and cancer risk	[34]
Protein Surrogates	EpiScores for plasma proteins	Plasma protein concentration	[35]

### 2.1. Epigenetic clocks

Epigenetic clocks are the most extensively developed DNAm-based predictors, including chronological age clocks, biological age clocks, and pace-of-aging clocks. These clocks capture aging-related changes that correlate with healthspan, lifespan, and disease risk.

Chronological Age Clocks are designed to estimate an individual’s calendar age. The Horvath Clock [8] is a widely used pan-tissue predictor with 353 CpGs and trained across 51 different tissue and cell types, while the Hannum Clock [9] is a blood-based model using 71 CpGs. To improve predictive accuracy, newer models have been developed using larger and more diverse datasets, including cAge by Bernabeu et al. [18] and Epigenetic Age by Zhang et al. [19]. For pediatric populations, the Pediatric Buccal Epigenetic (PedBE) clock [20] was specifically designed to estimate biological age in children and adolescents using buccal epithelial cells. To estimate the gestational age of a fetus or newborn, several specialized predictors have been developed, including GACPC, GARPC, and GARRPC based on placental tissue DNAm [21], as well as BohlinGAge [22] and KnightGAge [23], which were trained on cord blood DNA. More recently, deep learning-based models such as DeepMAge [16] and AltumAge [17] have been developed to enhance the accuracy and robustness of chronological age prediction across multiple tissues and platforms. Using DNAm markers as surrogates for metabolites, Xu et al. [24] developed a DNAm-metabolic clock by first predicting metabolite levels from DNAm data. This hybrid clock, trained on DNAm-derived metabolite proxies, predicts chronological age and is strongly associated with disability, gait speed, mortality, and disease risk, offering a novel approach to study metabolic aging using only methylation data.

Biological Age Clocks aim to capture the biological processes of aging that correlate with healthspan and disease risk, rather than just calendar time. These clocks are more strongly associated with age-related morbidity and mortality and are particularly useful for evaluating biological aging and the effects of interventions. The PhenoAge clock [10] integrates clinical biomarkers such as albumin, glucose, and CRP to construct a phenotypic age, while GrimAge [11] uses DNAm-based surrogates for plasma proteins and smoking history to construct a composite biomarker of lifespan. Other biological clocks include the Zhang10CpG model [25], which predicts all-cause mortality using a 10-CpG signature; the Frailty clock [26], which estimates a frailty index reflecting physiological decline; and bAge [18], an all-cause mortality trained biological age predictor based on epigenetic surrogates of plasma proteins and GrimAge component variables. Additionally, DNAmFitAge [27] integrates DNAm markers linked to physical fitness measures (gait speed, grip strength and VO₂max) along with GrimAge, providing a functional perspective of biological aging. As a related but distinct predictor, DNAmTL [28] estimates telomere length from DNAm data, providing insight into cellular replicative aging and potential age-related disease risk.

Pace-of-Aging Clocks estimate the rate at which an individual is aging biologically over time. A key example is DunedinPACE, developed from the longitudinal Dunedin Study [15,36], which measures systemic physiological decline across multiple organ systems (cardiovascular, metabolic, renal, hepatic, immune, dental, and pulmonary systems). Rather than providing a static age estimate, these clocks offer a dynamic assessment of aging velocity, making them especially valuable in clinical trials and longitudinal aging research.

### 2.2. Disease risk prediction

DNAm-based predictors can be used to assess disease risk, often before clinical symptoms appear. These models utilize either targeted CpG panels or poly-epigenetic risk scores, conceptually similar to polygenic risk scores, to estimate susceptibility to various diseases. In oncology, several clinically validated DNAm-based predictors have demonstrated promising utility for early detection and risk assessment. The Epi proColon® test is the first FDA-approved blood-based DNAm assay for colorectal cancer detection, targeting methylation at the *SEPT9* gene [29]. More recently, the Galleri® test, a multi-cancer early detection assay, analyzes cell-free DNAm signatures to identify over 50 types of cancer with high specificity [30]. Targeted methylation panels are also being developed for early detection and monitoring of breast and prostate cancer, showing promise for guiding personalized treatment strategies [37,38]. Additionally, methylation risk scores (MRS) developed from large population cohorts have shown predictive value for coronary artery disease, myocardial infarction, and stroke [31].

### 2.3. Lifestyle exposure biomarkers

DNAm can serve as a robust biomarker of cumulative environmental and lifestyle exposures, offering a molecular-level record of an individual’s exposome. One of the examples is smoking, where CpG sites in genes such as *AHRR*, *F2RL3*, and *GPR15* are consistently hypomethylated in both current and former smokers. Predictive models like EpiSmokEr [12] leverage these loci to generate accurate smoking status (never, former, or current) using 121 CpG sites. In contrast, McCartney et al. [13] developed a DNAm predictor that estimates cumulative smoking exposure in pack-years, providing a continuous measure useful for assessing lifetime risk in epidemiological studies.

Similarly, several studies have developed and validated DNAm-based predictors of alcohol consumption. Liu et al. [14] created a biomarker using 144 CpGs that accurately estimated alcohol use. McCartney et al. [13] developed a predictor using CpG sites associated with self-reported alcohol intake. The predictor demonstrated strong accuracy and was associated with mortality, making it a valuable tool for evaluating alcohol exposure in large-scale studies. Lohoff et al. [39] developed a DNAm-based risk score for alcohol use disorder using 519 CpG sites associated with alcohol consumption. The predictor offers insight into the biological pathways underlying alcohol use disorder and could support early detection of individuals at elevated risk.

### 2.4. Trait-specific DNAm predictors

Trait-specific DNAm predictors estimate a wide range of biological and clinical phenotypes, offering valuable tools for understanding individual variability in health and disease. These models are typically developed using penalized regression techniques, enabling robust trait imputation across populations.

Metabolic and anthropometric traits were among the first non-aging-related phenotypes predicted using DNAm data. Using large cohorts such as Generation Scotland, McCartney et al. [13] developed DNAm-based predictors for body mass index (BMI), body fat percentage, waist-to-hip ratio (WHR), and HDL cholesterol. These models leverage hundreds of trait-associated CpGs and show strong correlation with clinical measures, offering a means to assess cardiometabolic risk in large-scale studies where direct measurements may be missing or unreliable.

Physical function and fitness represent key domains of healthspan that can also be inferred from DNA methylation. McGreevy et al. [27] constructed a suite of DNAm-based predictors targeting grip strength (DNAmGrip_wAge, DNAmGrip_noAge), gait speed (DNAmGait_wAge, DNAmGait_noAge), lung function (DNAmFEV1_wAge), and cardiorespiratory fitness (DNAmVO2max). These predictors facilitate the assessment of physical function and frailty in aging populations and are integrated into the DNAmFitAge model to provide a biologically driven, exercise-responsive indicator of functional capacity.

Stem cell division and mitotic history predictors estimate the cumulative number of cell divisions within a tissue and are considered markers of replicative aging and proliferative stress. pcgtAge [32] captures methylation drift in polycomb group target (PCGT) CpGs that are unmethylated in fetal tissues and DNAm levels increase with age. MiAge Youn and Wang [33] uses mitotic informative CpGs to estimate stem cell turnover across tissues. TNSC and TNSC2 [34] provide refined measures of mitotic-like activity by capturing stochastic methylation variability and CpG island drift, with implications for both aging and cancer risk.

DNAm-based protein surrogates represent another rapidly growing area of trait prediction, enabling indirect estimation of plasma protein concentrations from methylation data. Gadd et al. [35] developed DNAm predictors for 109 proteins, covering a wide range of biological pathways including inflammation, metabolism, and neurodegeneration. While earlier models like GrimAge provided DNAm-based proxies for a select number of proteins (e.g., PAI-1, GDF15), this expanded catalog enables large-scale multi-omic studies and functional annotation of epigenetic signatures when proteomic data are unavailable.

## 3. Methodological considerations

### 3.1. Methods and models for deriving DNAm predictors

A wide range of statistical and machine learning approaches have been used to construct DNAm-based predictors. These methods are designed to extract biologically meaningful patterns from high-dimensional methylation data while balancing predictive accuracy, interpretability, and robustness across technical and biological contexts.

One of the most widely adopted methods is penalized regression, particularly elastic net, which performs simultaneous variable selection and regularization. This approach underpins many foundational predictors, including Horvath and Hannum clocks for chronological age, and biological age models like PhenoAge and GrimAge. Penalized regression has also been employed to develop DNAm surrogates for clinical and biochemical traits such as BMI, body fat percentage, HDL cholesterol, and plasma proteins (e.g., PAI-1, GDF15, TIMP1), as well as behavioral traits like smoking pack-years. These models are typically trained on large, well-phenotyped datasets and have shown strong performance across cohorts.

In addition, machine learning and deep learning techniques have been increasingly applied to capture complex, non-linear relationships in DNAm data. Models like DeepMAge and AltumAge use deep neural networks trained on large multi-cohort datasets to improve age prediction accuracy across tissues and platforms. While these methods offer enhanced predictive power, particularly for complex biological traits, they make it difficult to track the contributions of individual CpG sites to the final prediction and require more extensive computational resources and tuning.

Another modeling strategy involves the use of surrogate markers, in which DNAm is used to estimate unmeasured traits such as protein levels or physiological performance metrics. This approach was notably implemented in GrimAge, which first derives DNAm surrogates for intermediate traits and then combines them into a composite mortality predictor. Similar frameworks have been used to model DNAmFitAge. These models are particularly useful for integrating biological function into DNAm-based health assessments.

Longitudinal models offer a dynamic view of aging by estimating the rate of biological change over time rather than a static trait level. DunedinPACE, for example, was developed using two decades of repeated phenotyping in the Dunedin Study and quantifies the pace of physiological decline across multiple systems [15]. These models are especially valuable in aging intervention research and life-course studies.

Another class of models captures methylation drift and stochastic epigenetic variability to reflect stem cell division and mitotic history. Predictors such as pcgtAge, MiAge, TNSC, and TNSC2 estimate replicative aging by modeling variability at CpG sites linked to mitotic activity, including sites within or near Polycomb Group Target (PCGT) promoters and regions prone to age-related methylation changes. These models, developed primarily through feature selection based on methylation variability rather than supervised regression, are designed to be generalizable across multiple tissues. They offer insights into tissue turnover, transcriptional instability, and cancer susceptibility, providing a complementary dimension to traditional measures of chronological and biological aging.

To reduce measurement noise and technical variability, Higgins-Chen et al. [40] introduced a principal component (PC)-based framework to enhance the reliability of DNAm predictors. Instead of training models on CpG beta values, they used principal component scores derived from the beta matrix. This approach was also applied to reconstruct individual DNAm surrogates in GrimAge (e.g., PCPAI1, PCGDF15, PCADM, PCLeptin, PCCystatinC, PCTIMP1, PCB2M, PCPACKYRS). The PCA-based method substantially improved test – retest reliability and reduced batch effects, particularly in longitudinal and cross-platform settings, while preserving biological relevance.

### 3.2. Data preprocessing and normalization

Robust and reproducible DNAm-based predictors require not only well-constructed models but also careful attention to data preprocessing and normalization. Differences in preprocessing pipelines can significantly affect predictor performance, potentially introducing bias in cross-cohort or longitudinal analyses. To evaluate this, Ori et al. [41] systematically assessed the impact of various preprocessing strategies on over 40 established DNAm predictors, including GrimAge, PhenoAge, and smoking scores. Using a large, well-characterized dataset, they compared popular pipelines such as minfi, watermelon and ENmix, measuring performance based on correlation with chronological age, associations with health outcomes, and between-batch consistency. While no single approach proved optimal for every predictor, ENmix consistently delivered the best overall performance, demonstrating high accuracy and strong reproducibility across batches.

## 4. Software and tools for calculating DNAm predictors

A wide range of software tools have been developed to facilitate the calculation of DNAm based health predictors. Most of these tools were designed to implement individual predictors using model weights or coefficients published by their original developers. While effective, these single-purpose tools often required users to navigate multiple software environments, data preprocessing standards, and input formats – creating barriers to reproducibility and cross-study comparability. To overcome these limitations, several comprehensive toolkits have emerged, enabling the standardized and streamlined computation of multiple DNAm predictors with a unified analytical framework.

Among the most widely used integrated tools are methylclock [42], Horvath’s DNAmAge online calculator [8], and methscore [43]. The R package methylclock supports the computation of over a dozen well-established clocks, including Horvath, Hannum, PhenoAge, GrimAge, DNAmTL and PedBE. Horvath’s DNAmAge online calculator offers a web-based interface for users without programming experience, allowing for the upload of preprocessed beta value files and returning multiple epigenetic age estimates and related metrics. The methscore is a comprehensive function within the ENmix R package. Specifically designed to unify and standardize the calculation of DNAm-based predictors, it enables the computation of more than 150 predictors, such as chronological and biological age, environmental and lifestyle exposures, and DNAm-based surrogates for serum protein levels. It incorporates a reference-based normalization approach to adjust for batch and array differences, account for missing CpGs and thereby improving consistency across diverse datasets.

## 5. Clinical and research applications

DNAm based health predictors are increasingly transitioning from research into real-world applications, including clinical diagnostics, intervention trials, and public health monitoring. Their sensitivity, noninvasive sampling, and biological relevance make them promising tools for early disease detection and tracking responses to therapies or lifestyle changes. For example, cell free DNAm-based tests like Galleri® can detect over 50 cancers with high specificity [30], while others such as Epi proColon® are FDA-approved for colorectal cancer screening [29]. Methylation markers are also being explored for lung, bladder, and prostate cancers [37,38,44], as well as for cardiometabolic conditions using methylation profile scores [31].

In aging research, epigenetic clocks are used as surrogate biomarkers in trials aiming to slow or reverse biological aging. Studies involving senolytic drugs, hormonal therapies, or caloric restriction have reported reductions in DNAm-derived age estimates [45–48]. Recent advances in clock design, such as DunedinPACE and supervised PCA clocks, have improved sensitivity and technical robustness, enhancing their utility in intervention trials [15,40].

## 6. Challenges and future directions

Despite their growing promise, DNAm-based health predictors face technical, biological, and interpretive challenges that must be addressed to ensure reliability, equity, and clinical utility.

Reproducibility remains a major limitation. DNAm data are vulnerable to batch effects, platform differences, and variability in preprocessing pipelines such as ENmix, minfi, and wateRmelon. Even widely used clocks like GrimAge and PhenoAge can yield inconsistent results across different workflows [41].

Tissue specificity is another constraint. Most predictors are trained on blood-derived DNAm data, which may not capture disease-relevant changes occurring in other organs such as the brain or liver [49].

Interpretation challenges persist. While many CpGs correlate with aging and disease, causal relationships are often unclear. Methylation changes may be downstream effects rather than upstream drivers, complicating their use for early diagnostics. Although tools like Mendelian randomization (MR) aim to infer causality, they are limited by tissue specificity, the availability of robust mQTLs, and methodological complexity. Furthermore, the timing of DNAm changes relative to disease onset is poorly understood, raising concerns about reverse causality.

Population generalizability is another pressing issue. Most DNAm predictors have been developed in cohorts of European ancestry, leading to reduced accuracy in diverse ethnic and socio-economic groups [50]. Overfitting, limited external validation, and sensitivity to transient physiological states such as inflammation further constrain broader adoption. Addressing these limitations will require larger, more diverse datasets and improved validation in real-world settings.

As DNAm predictors advance toward clinical use, ethical and equity considerations become increasingly important. Epigenetic data can reveal sensitive information about environmental exposures and aging, raising issues around privacy, re-consent, and incidental findings [51]. Existing legal protections may be insufficient to prevent the misuse of epigenetic age estimators in contexts such as insurance underwriting or employment decisions [52].

Looking ahead, the field is advancing toward more dynamic, integrative, and personalized applications. Multiomics integration, combining DNA methylation with transcriptomic, proteomic, and metabolomic data, offers a more comprehensive view of health and disease. Predictors like GrimAge incorporate DNAm surrogates for plasma proteins, and a recent study developed a hybrid DNAm – metabolic clock using DNAm as surrogates for metabolites [24]. At the same time, artificial intelligence is transforming the field. Large-scale pretrained models like CpGPT, trained on hundreds of DNAm datasets, can generalize across tasks without retraining [53], while other models, such as AltumAge and DeepMAge, utilize deep learning to enhance age prediction accuracy. Finally, a shift toward longitudinal and dynamic tracking is redefining applications of DNAm predictors. Clocks like DunedinPACE and PoAm estimate the pace of aging, enabling monitoring of health trajectories and responses to interventions [15].

The vast majority of DNAm-based predictors described in this review were developed using Illumina 450K or EPIC BeadChip arrays, which are cost-effective and widely used for epigenome-wide association studies (EWAS). With advancements in technology and reduced costs, resequencing methods like whole-genome bisulfite sequencing (WGBS), reduced representation bisulfite sequencing (RRBS), and genome-wide nanopore sequencing offer higher resolution but pose challenges for applying array-based predictors. These include ensuring overlap of predictor-specific CpG probes and normalizing data for comparable methylation measures. Comprehensive evaluations are needed to assess the applicability of existing predictors to resequencing data or develop data-type-specific predictors for diverse sequencing platforms.

## 7. Conclusion

DNAm-based predictors are rapidly transforming how we quantify biological aging, assess disease risk, and track environmental and lifestyle exposures. Their sensitivity to dynamic physiological states, combined with their noninvasive and scalable nature, makes them powerful tools across both research and clinical domains. As these predictors evolve from chronological age estimators to complex multi-trait biomarkers, including disease risk, physical function, and environmental exposures, they offer a uniquely integrated view of health.

Despite their potential, several barriers hinder broader clinical applications. Technical variability introduced by array platforms, batch effects, and inconsistent preprocessing workflows continues to undermine reproducibility across studies and populations. Tissue specificity and unclear causal directionality further complicate interpretation, while the underrepresentation of non-European ancestry groups in training datasets raises critical concerns about generalizability and equity. At the same time, the ethical dimensions of epigenetic data, particularly privacy, consent, and potential misuse, demand proactive regulation and transparent governance.

Recent advances offer promising solutions. The integration of methylation with other omics data, such as transcriptomics, proteomics, and metabolomics, is yielding more biologically informed and tissue-relevant predictors. Deep learning models like DeepMAge and AltumAge, and AI powered tools such as CpGPT, demonstrate improved adaptability across populations and tissues. Tools like methscore and principal component-based frameworks also enhance reproducibility, enabling more reliable longitudinal applications.

To fully realize the promise of DNAm biomarkers, future efforts must prioritize inclusive cohort development, harmonized preprocessing pipelines, and robust validation across diverse settings. Just as important is the establishment of ethical frameworks that support responsible data use and equitable access. If these scientific and societal challenges are addressed, DNAm-based predictors could become valuable tools in precision medicine, enabling earlier interventions, personalized health monitoring, and a deeper understanding of the biological mechanisms underlying human aging and disease.

### 7.1. Future perspective

Over the next 5–10 years, the field of DNAm-based predictors, or methylation clocks, is poised to undergo significant advancements driven by technological innovations, expanded datasets, and interdisciplinary integration. Improvements in high-throughput sequencing and single-cell methylation profiling will likely enable the development of more precise and tissue-specific predictors. Machine learning models, including deep learning, are expected to enhance predictive accuracy by capturing complex epigenetic patterns. The integration of multi-omics data, such as transcriptomics and proteomics, will facilitate the development of holistic biomarkers for biological aging, chronological age, and disease risk. Additionally, the expansion of diverse, global cohorts will enhance the generalizability of DNAm predictors across populations. Emerging user-friendly software tools will foster broader adoption of methylation clocks in research and diagnostics. However, challenges like data privacy and ethical issues in personalized medicine will require robust guidelines to ensure responsible use of DNAm-based predictors. We anticipate that collaborative efforts between epigenomics researchers, data scientists, and clinicians will lead to more reliable, equitable, and interpretable predictors, transforming their role in precision health over the coming decade.

## Article highlights

- Comprehensive review of DNA methylation-based predictors, categorizing their applications in chronological age, biological aging, disease risk, and lifestyle exposures.
- Details development methods and characteristics of DNAm predictors, aiding researchers in selecting tools based on data type and study goals.
- Discusses software tools like methylclock and methscore for calculation of DNAm predictors.
- Evaluates challenges in model interpretability, overfitting risks, and validation across diverse populations, with mitigation strategies.
- Highlights future directions for improving reproducibility and generalizability of DNAm predictors in epigenomics.
